# Increased abundance of actinobacteria and upregulation of primary bile acid biosynthesis in diabetic foot ulcers

**DOI:** 10.3389/fcimb.2025.1651828

**Published:** 2025-11-20

**Authors:** Mi Zhou, Han Zhang, Rui Zhang, Tianshu Wei, Xiaojun Zhou

**Affiliations:** 1 Department of Vascular Surgery, Beijing Jishuitan Hospital, Capital Medical University, Beijing, China; 2 Department of Endocrinology and Metabology, The First Affiliated Hospital of Shandong First Medical University & Shandong Provincial Qianfoshan Hospital, Shandong Key Laboratory of Rheumatic Disease and Translational Medicine, Shandong Institute of Nephrology, Jinan, Shandong, China; 3 Phase I Drug Clinical Trial Center, Qilu Hospital of Shandong University, Jinan, China; 4 Clinical Laboratory, The First Affiliated Hospital of Shandong First Medical University & Shandong Provincial Qianfoshan Hospital, Jinan, Shandong, China; 5 State Key Laboratory of Discovery and Utilization of Functional Components in Traditional Chinese Medicine, School of Pharmaceutical Sciences, Cheeloo College of Medicine, Shandong University, Jinan, Shandong, China; 6 Drug Discovery Biology, Monash Institute of Pharmaceutical Sciences, Monash University, Parkville, VIC, Australia

**Keywords:** diabetic foot, 16S rRNA sequencing, microbiota, atherosclerosis, actinobacteria, primary bile acid biosynthesis

## Abstract

**Background:**

Vascular microbial imbalance may disrupt homeostasis and impair wound healing by triggering local and systemic inflammation. Diabetic foot (DF), a severe complication of diabetes, is frequently associated with bacterial infections. However, the arterial microbiota in DF remains unexplored. This study characterizes the arterial microbiota in DF patients and investigates its potential role in disease progression.

**Methods:**

A total of 22 patients were recruited, including those undergoing surgery for DF, patients with lower limb atherosclerosis (AS) requiring surgery, and trauma patients who underwent amputation as healthy controls (C). Samples were obtained under sterile conditions, and 16S rRNA sequencing was performed. Microbial diversity and functional pathways were analyzed using QIIME2.

**Results:**

Alpha diversity analysis revealed a progressive decline in microbial diversity from the C group to the AS and DF groups. Beta diversity analysis demonstrated that the DF and AS groups clustered closely, while both exhibited significant microbial compositional differences compared to the C group (ANOSIM, *P* < 0.01). At the phylum level, Actinobacteria was significantly enriched in the DF and AS groups, whereas TM6 was reduced in the DF group and Proteobacteria was reduced in the AS group. LEfSe analysis identified *Corynebacterium*, *Streptophyta_Group*, *Caulobacter*, *Hydrogenophaga*, and *Diaphorobacter* as key representative genera in the DF group. Furthermore, KEGG analysis revealed metabolic alterations in both the DF and AS groups, including upregulated metabolism and organismal system pathways. At level 3, the DF group exhibited significant downregulation of amino sugar and nucleotide sugar metabolism, whereas primary bile acid biosynthesis was markedly upregulated, suggesting a potential role in DF progression.

**Conclusion:**

DF is associated with distinct alterations in arterial microbiota composition and metabolic pathways. Alterations in Actinobacteria and primary bile acid biosynthesis may be relevant to DF progression and could serve as potential therapeutic targets.

## Introduction

Diabetic Foot (DF) is a common and severe complication of diabetes, accompanied by a high risk of amputation (Chen et al., 2023). The pathogenesis of DF begins with poorly managed blood sugar levels and involves the interaction between angiopathy, neuropathy, and infection (Liu et al., 2021). Atherosclerosis (AS), as a key pathological basis of DF, significantly impacts the occurrence and deterioration of DF (Deng et al., 2023). Prolonged hyperglycemia damages vascular endothelial function (Huang et al., 2020) while triggers abnormalities in hemodynamics, stimulating the thickening of blood vessel walls and the formation of atherosclerotic plaques (Yuan et al., 2019). As a result, blood supply to the lower extremities becomes restricted, leading to tissue hypoxia and metabolic disruptions. Meanwhile, it provides a pathological basis for the development and infection of DF ulcers. Rapidly expanding deep infections in DF ulcers often brings a significant challenge to clinical management (Lipsky et al., 2020). Despite the gradual recognition of the role of atherosclerosis in DF, the precise pathogenesis of DF requires further clarification.

Microorganisms play a crucial role in the pathogenesis of DF as causative agents of infections. Current literature predominantly focuses on the microbial flora found in DF ulcers (Jnana et al., 2020). The majority of such research adopt direct sampling methods, which are highly susceptible to contamination lack standard operating procedure. Hence, current literature remains inadequate in guiding pathogen culture and the use of antibiotics in DF (Du et al., 2022). Notably, microorganisms involved in DF are not limited to the ulcer sites. Changes in vascular microbiota may provide new insights into the identification of pathogenic organisms in DF infections (Koren et al., 2011). Microbiota have been discovered within the blood vessel and are expected to be key players in the development of DF. Emerging evidence suggests that multiple microorganisms, especially bacteria, reside in atherosclerotic plaques (Muhlestein, 2000; Ross, 1999). These organisms promote the development of atherosclerosis by inducing inflammatory responses (Brandsma et al., 2019; Shi et al., 2022; Voronina and Arapidi, 2024). Diabetic vascular complications are primarily characterized by endothelial dysfunction and atherosclerosis (Li et al., 2023). Given that atherosclerosis shares pathogenic features with DF, it is important to explore the characteristics and roles of microorganisms within blood vessels in the context of DF.

This study investigates the differences in arterial microbiota among DF patients, AS patients, and healthy controls through 16S rRNA gene sequencing (Fuks et al., 2018). Significantly different from the control group, both DF group and AS group demonstrated increased abundance of Actinobacteria. Linear Discriminant Analysis Effect Size (LEfSe) analysis revealed that *Corynebacterium*, *Streptophyta_Group*, *Caulobacter*, *Hydrogenophaga*, and *Diaphorobacter* are representative bacterial genera in the DF group. Furthermore, Kyoto Encyclopedia of Genes and Genomes (KEGG) pathway analysis indicated significant upregulation of primary bile acid biosynthesis, while amino sugar and nucleotide sugar metabolism were significantly downregulated in the DF group. Targeting relevant microbiota and metabolic pathways may offer new avenues for improving the prognosis of DF, providing novel directions for its treatment.

## Methods

### Sample collection

Arterial tissue samples for this study were obtained from clinical collections conducted at Beijing Jishuitan Hospital, Capital Medical University (Beijing, China). A total of 22 arterial tissue samples were collected, including 11 lower limb arterial samples from DF patients, 7 from AS patients, and 4 from healthy individuals who underwent amputation due to trauma, with their demographic characteristics presented in **Supplementary Table 1**. Considering that collecting completely healthy lower limb arteries would be ethically infeasible, control arterial tissues were obtained within 6 hours post-trauma. This study was approved by the Ethics Committee of Beijing Jishuitan Hospital, and informed consent was obtained from all participants. All surgical procedures were performed under strict sterile condition. The arterial tissues were acquired from femoral and popliteal arteries. Samples were immediately frozen in liquid nitrogen after surgery for subsequent analysis.

### Bacterial DNA extraction and sequencing

Microbial DNA was extracted from each sample using the cetyltrimethylammonium bromide (CTAB) method. The total microbial DNA was then quantified using a Qubit fluorometer (Invitrogen, USA). To ensure sequencing accuracy, multiple hypervariable regions of the 16S rRNA gene were amplified.

The reaction system of polymerase chain reaction (PCR) amplification was performed in a reaction system containing 12.5 μL of Phusion^®^ Hot Start Flex 2X Master Mix, 2.5 μL of primers, 50 ng of sample DNA, and ddH_2_O to a final volume of 25 μL. The PCR conditions were as follows: an initial denaturation at 98 °C for 30 s, followed by 30 cycles of denaturation at 98 °C for 10 s, annealing at 62 °C for 15 s, and extension at 72 °C for 35 s, with a final extension at 72 °C for 5 min. The amplification products were purified using AMPure XT magnetic beads to obtain high-quality PCR products.

A second round of PCR was performed to further amplify five hypervariable regions (V2, V3, V5, V6, and V8) of the 16S rRNA gene. The purified PCR products were evaluated using an Agilent 2100 Bioanalyzer (Agilent, USA) and a library quantification kit from Illumina (Kapa Biosciences, Woburn, MA, USA). The qualified libraries were then serially diluted, pooled, and denatured with NaOH to generate single-stranded DNA for sequencing. Paired-end sequencing (PE150) was conducted on the Illumina NovaSeq 6000 platform using the NovaSeq 6000 SP reagent kit (500 cycles).

### Sequencing data processing

The sequencing data were processed through a series of steps, including the removal of low-quality sequences, barcode sequence trimming, and primer removal. Quality filtering was performed using fqtrim software, where reads with quality scores below 20 were truncated, and sequences shorter than 100 bp were discarded.

Sequences from the five amplified regions were integrated for analysis, and taxonomic annotation was performed using the SILVA 138 database. Amplicon Sequence Variants (ASVs) were identified to ensure high-resolution microbial profiling. Alpha and beta diversity analyses were conducted using QIIME2 to compare microbial diversity among different groups.

### Contamination control measures

Given the low biomass of arterial tissue samples, strict contamination control was implemented throughout the workflow. During sample processing, extraction blanks, PCR negative controls, and sequencing blanks were included, and these controls underwent the same sequencing and analysis procedures as the study samples. The number of sequences detected in the controls was very low. For sequence processing, the DADA2 pipeline was used, incorporating stringent error correction and chimera removal steps to further minimize potential technical contamination.

### LEfSe analysis

The Linear Discriminant Analysis Effect Size (LEfSe) tool was used to identify significant microbial biomarkers among different groups. A linear discriminant analysis (LDA) threshold of >3 and a p-value <0.05 were set as criteria for selecting differentially enriched microorganisms in the DF group. These identified microbes serve as potential candidate biomarkers for DF.

### Functional prediction of microbiota

The functional potential of microbial communities was predicted based on 16S rRNA sequencing data using PICRUSt2 (Phylogenetic Investigation of Communities by Reconstruction of Unobserved States). Welch’s t-test was applied to identify functional differences between groups, and significantly different gene functions were annotated using the Kyoto Encyclopedia of Genes and Genomes (KEGG) database.

## Results

### Amplicon sequence variants and sequencing depth analysis

**Figure 1:**
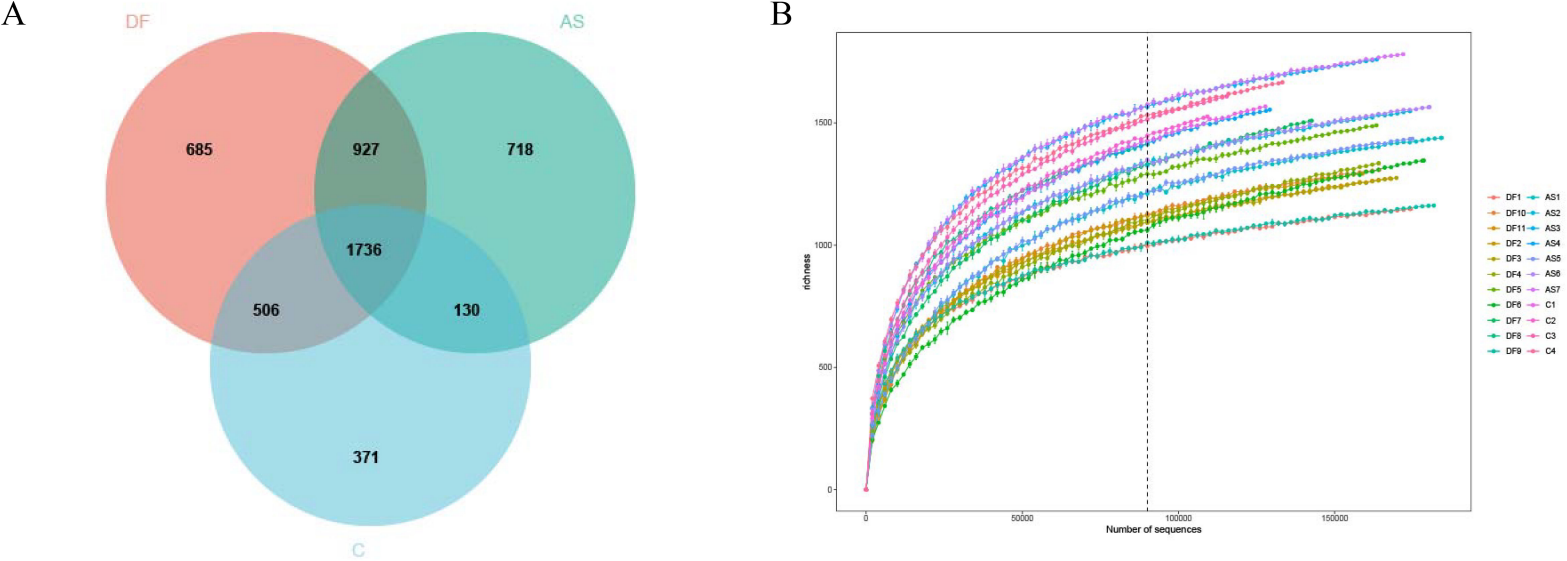
Venn diagram and rarefaction curve **(A)** Venn diagram. Each circle represents a group, with overlapping areas indicating the number of shared amplicon sequence variants (ASVs) between groups. Non-overlapping areas represent unique ASVs within each group. **(B)** Rarefaction Curve. The x-axis represents the number of randomly selected sequencing reads, while the y-axis indicates the number of ASVs constructed based on the sequencing depth. Different groups are represented by curves of different colors.

A total of 3,854 amplicon sequence variants (ASVs) were identified in this study, spanning 54 phyla, 121 classes, 216 orders, 376 families, and 861 genera. A Venn diagram was constructed to illustrate the distribution of ASVs among the three groups (**Figure 1A**), showing that 1,746 ASVs were shared across all samples. The DF group contained 3,854 ASVs, the AS group had 3,511 ASVs, and the C group had 2,743 ASVs. Specifically, 684 unique ASVs were found in the DF group, 718 in the AS group, and 371 in the C group. Rarefaction curves for the three groups approached a plateau (**Figure 1B**), indicating that the sequencing depth was sufficient.

### Reduced vascular microbial diversity and significant community variations in diabetic foot patients

**Figure 2:**
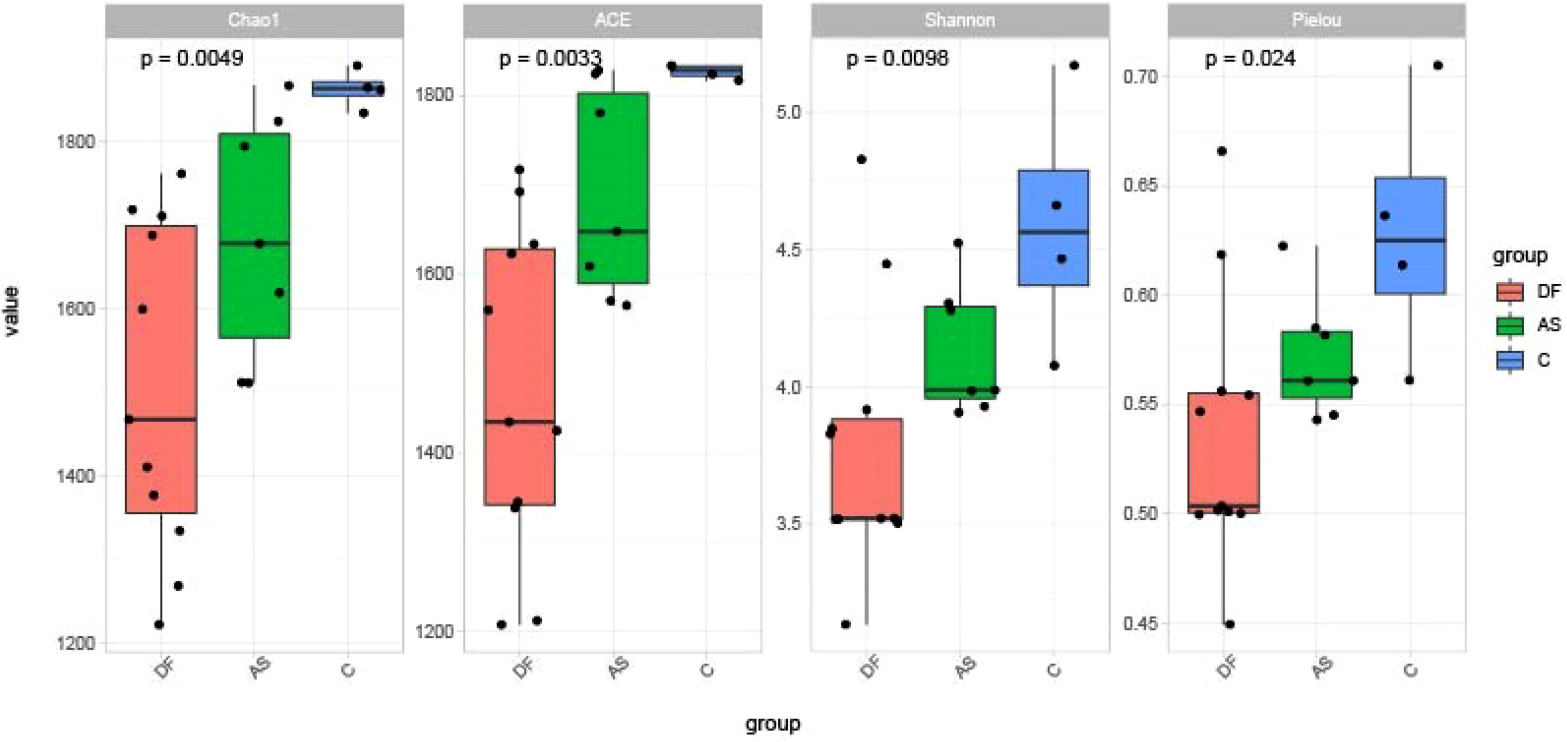
Alpha diversity. Comparisons of the Shannon index, Pielou index, Chao1 index, and ACE index among the three groups. All indices showed significant differences among groups (P < 0.05).

The alpha diversity among the three groups is shown in **Figure 2**. The diversity, richness, and evenness of species showed significant differences between the DF (*P* < 0.01), AS, and C groups, with the DF group having the lowest and the C group the highest.

**Figure 3:**
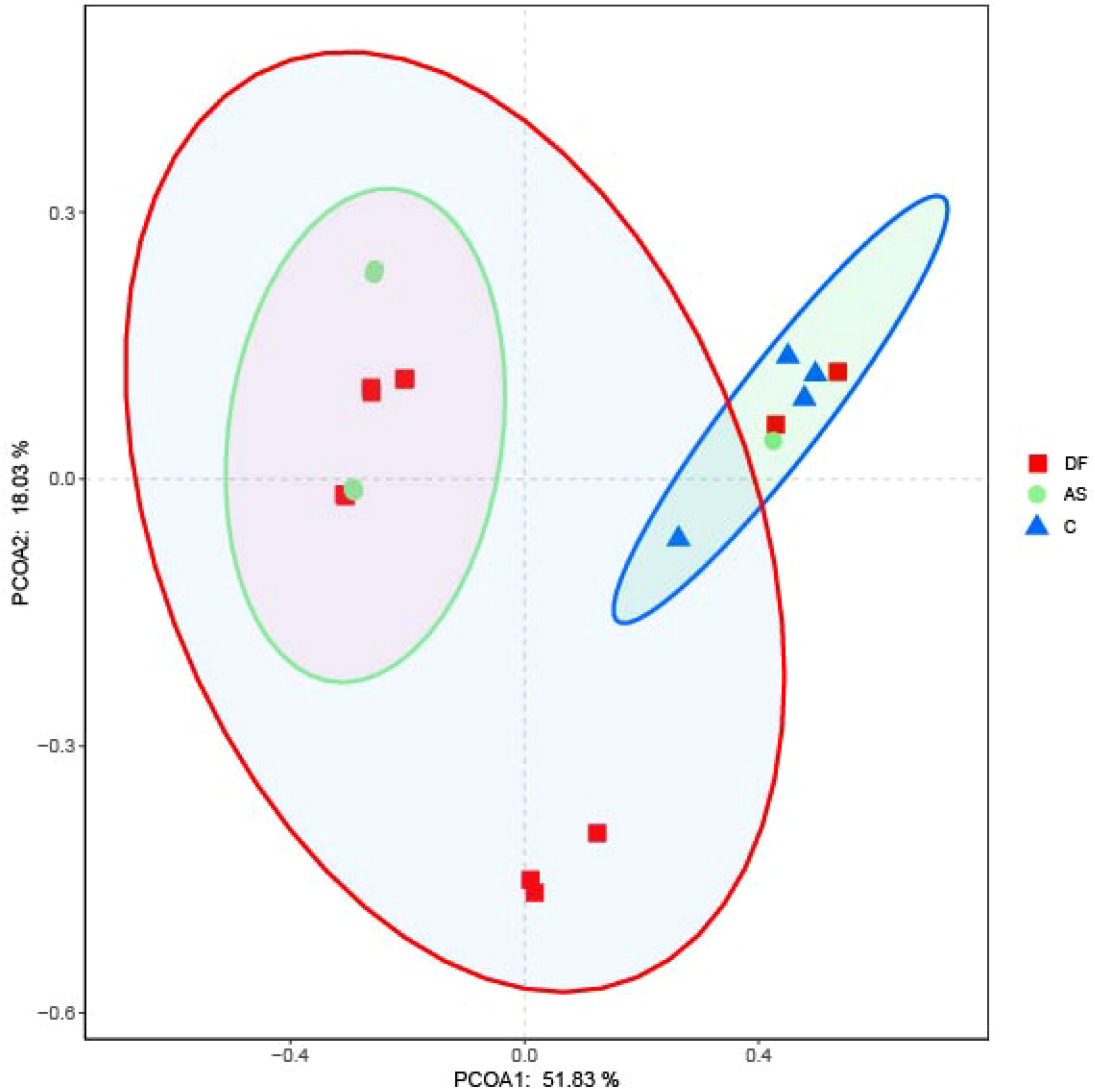
PCoA analysis. Principal coordinates analysis (PCoA) plot showing the distribution of samples. Samples from the same group are represented by the same color, with each point corresponding to an individual sample. The distance between points reflects the degree of dissimilarity. PCoA1 accounts for the greatest variation (51.83%), followed by PCoA2 (18.03%).

The beta diversity among the three groups is shown in **Figure 3**. Principal Coordinate Analysis (PCoA) based on the weighted UniFrac distance demonstrated that the samples in the DF and AS groups clustered more closely, suggesting that the bacterial evolutionary divergence between these two groups was similar, and their microbial community structures were also alike. In contrast, the C group was more distantly positioned from both the DF and AS groups, indicating a significant difference in bacterial community composition between the disease groups (DF and AS) and the healthy controls (C).

According to ANOSIM analysis, the composition of microbiota was not significantly different between the DF and AS groups *(P* = 0.525). However, both the DF and AS groups exhibited significantly different bacterial compositions compared to the C group (*P* < 0.05).

### Dysbiosis of the microbial community is present in the lower limb arteries of DF patients

**Figure 4:**
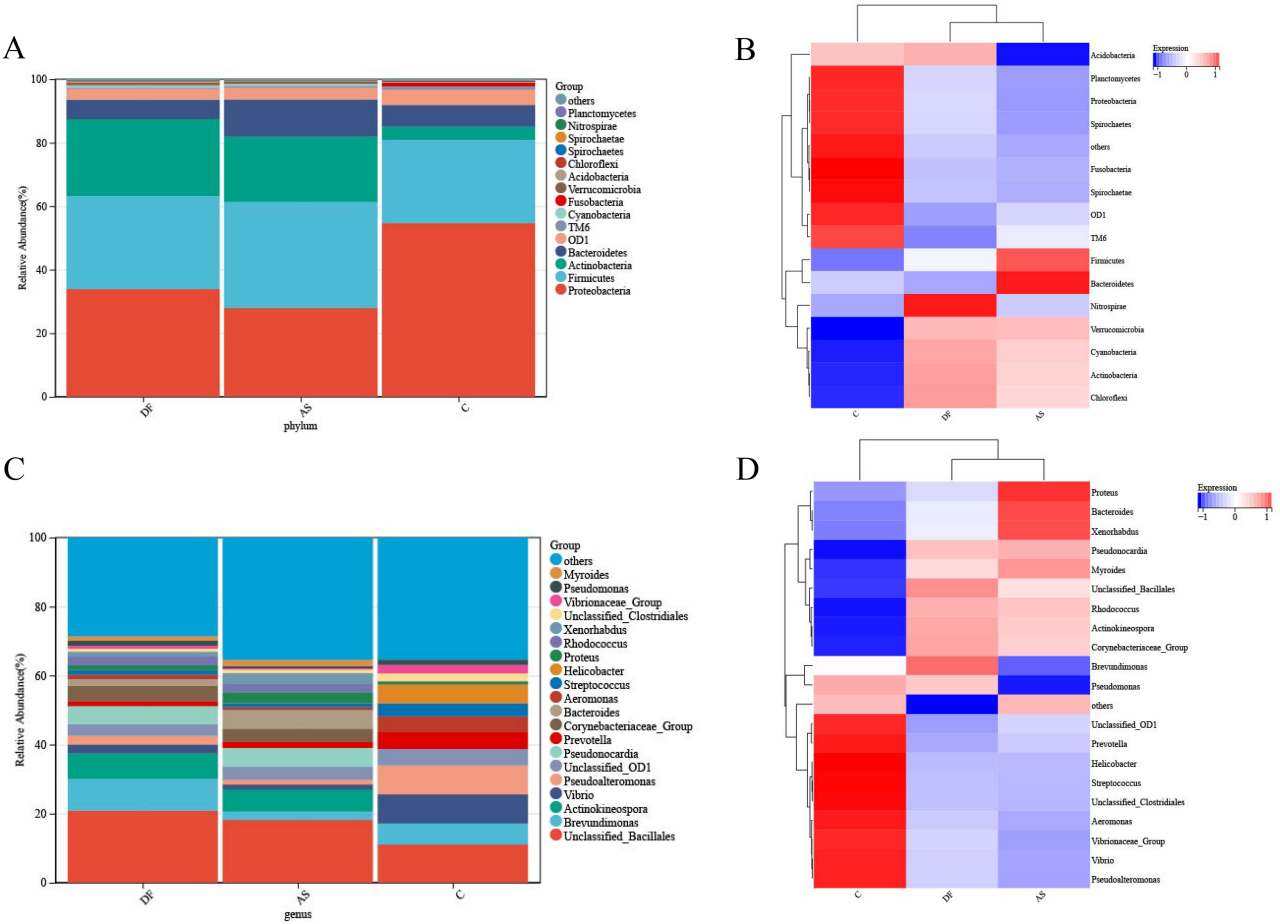
Relative abundance of microbial species. **(A)** Bar plot of relative abundance at the phylum level. **(B)** Heatmap of relative abundance at the phylum level. **(C)** Bar plot of relative abundance at the genus level. **(D)** Heatmap of relative abundance at the genus level.

At the phylum level, the microbiota in the arteries of the DF, AS, and C groups were predominantly composed of Proteobacteria and Firmicutes (**Figure 4A**). A cluster heatmap was used to display the relative abundance distribution at the phylum level (**Figure 4B**).

The DF group was primarily dominated by Proteobacteria, Actinobacteria, Firmicutes, Bacteroidetes, OD1, and Cyanobacteria, while the AS group was mainly dominated by Firmicutes, Proteobacteria, Actinobacteria, Bacteroidetes, OD1, Cyanobacteria, and TM6. The C group was dominated by Proteobacteria, Firmicutes, Bacteroidetes, OD1, Actinobacteria, Fusobacteria, and TM6.

The microbial composition in the DF group showed some similarities with the AS group, as both had a significantly higher abundance of Actinobacteria compared to the C group. However, the DF group had a significantly lower abundance of TM6, while the AS group had a significantly lower abundance of Proteobacteria compared to the C group.

At the genus level (**Figure 4C**), the microbiota in the arteries of the DF, AS, and C groups were predominantly composed of *Unclassified_Bacillales*. A cluster heatmap was used to display the relative abundance distribution at the genus level (**Figure 4D**).

The DF group was primarily dominated by *Unclassified_Bacillales*, *Brevundimonas*, *Actinokineospora*, *Pseudonocardia*, *Corynebacteriaceae_Group*, and Unclassified_OD1. The AS group was mainly dominated by *Unclassified_Bacillales*, *Actinokineospora*, *Pseudonocardia*, Bacteroides, *Corynebacteriaceae_Group*, and *Unclassified_OD1*. The C group was dominated by *Unclassified_Bacillales*, *Vibrio*, *Pseudoalteromonas*, *Brevundimonas*, *Helicobacter*, *Prevotella*, *Unclassified_OD1*, *Aeromonas*, and *Streptococcus*.

*Corynebacterium*, *Streptophyta_Group*, *Caulobacter*, *Hydrogenophaga*, and *Diaphorobacter* are the primary representative bacteria of the DF patients.

**Figure 5:**
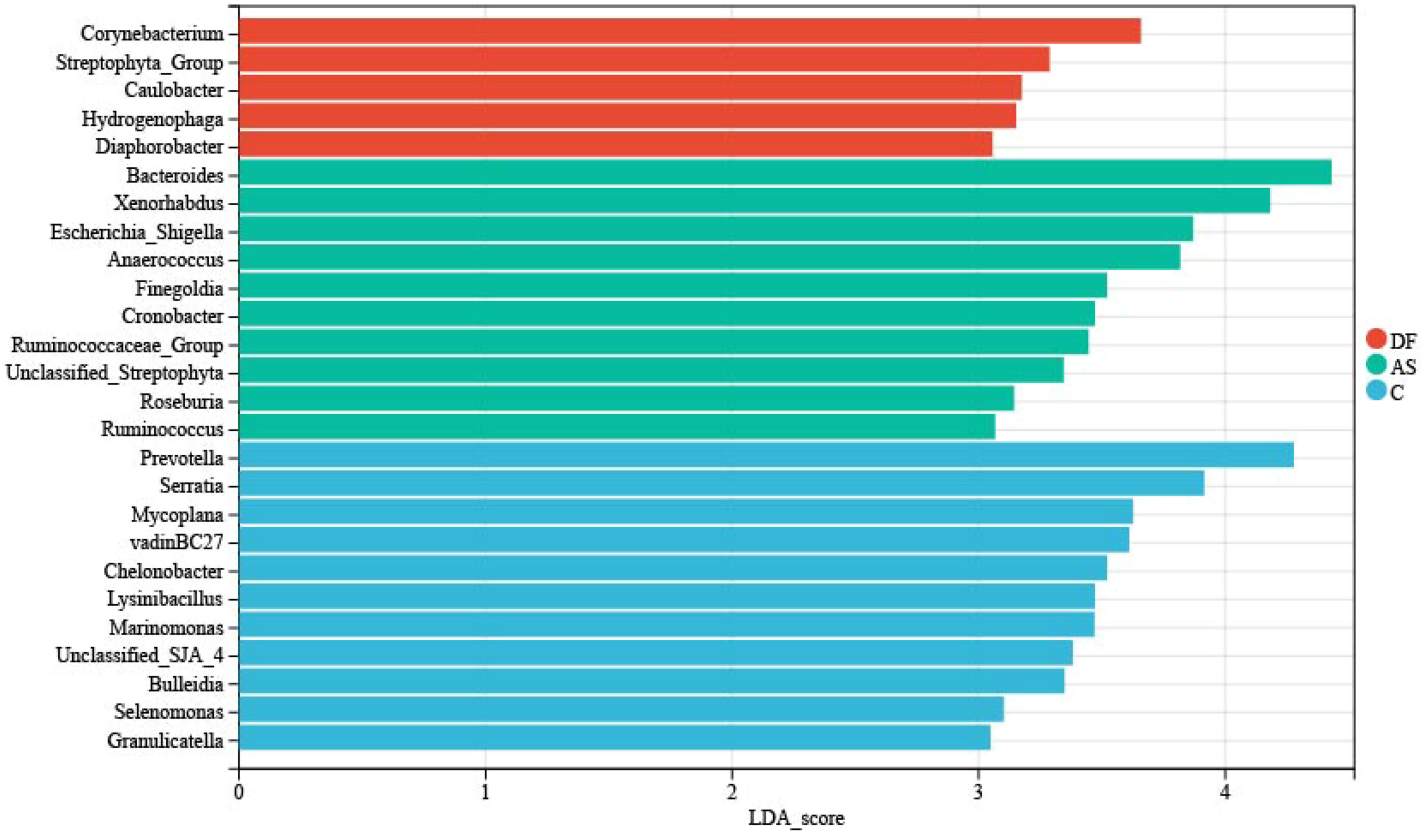
LEfSe analysis. The linear discriminant analysis (LDA) effect size (LEfSe) bar chart highlights significant biomarkers in the microbiome. The threshold for LEfSe analysis was set at LDA score > 3 and P < 0.05.

To further explore meaningful biomarkers, we conducted LEfSe analysis (**Figure 5**). The primary representative bacteria of the DF group were identified as *Corynebacterium*, *Streptophyta_Group*, *Caulobacter*, *Hydrogenophaga*, and *Diaphorobacter*. The main representative bacteria of the AS group were *Bacteroides*, *Xenorhabdus*, *Escherichia_Shigella*, *Anaerococcus*, *Finegoldia*, *Cronobacter*, *Ruminococcaceae_Group*, *Unclassified_Streptophyta*, *Roseburia*, and *Ruminococcus*. The primary representative bacteria of the C group were *Prevotella*, *Serratia*, *Lysinibacillus*, *vadinBC27*, *Mycoplana*, *Chelonobacter*, *Marinomonas*, *Unclassified_SJA_4*, *Bulleidia*, and *Selenomonas*.

### Primary bile acid biosynthesis is elevated in the lower limb arteries of DF patients

Using the PICRUSt algorithm, we performed functional predictions based on the Kyoto Encyclopedia of Genes and Genomes (KEGG) database for the microbial communities in the three groups.

**Figure 6:**
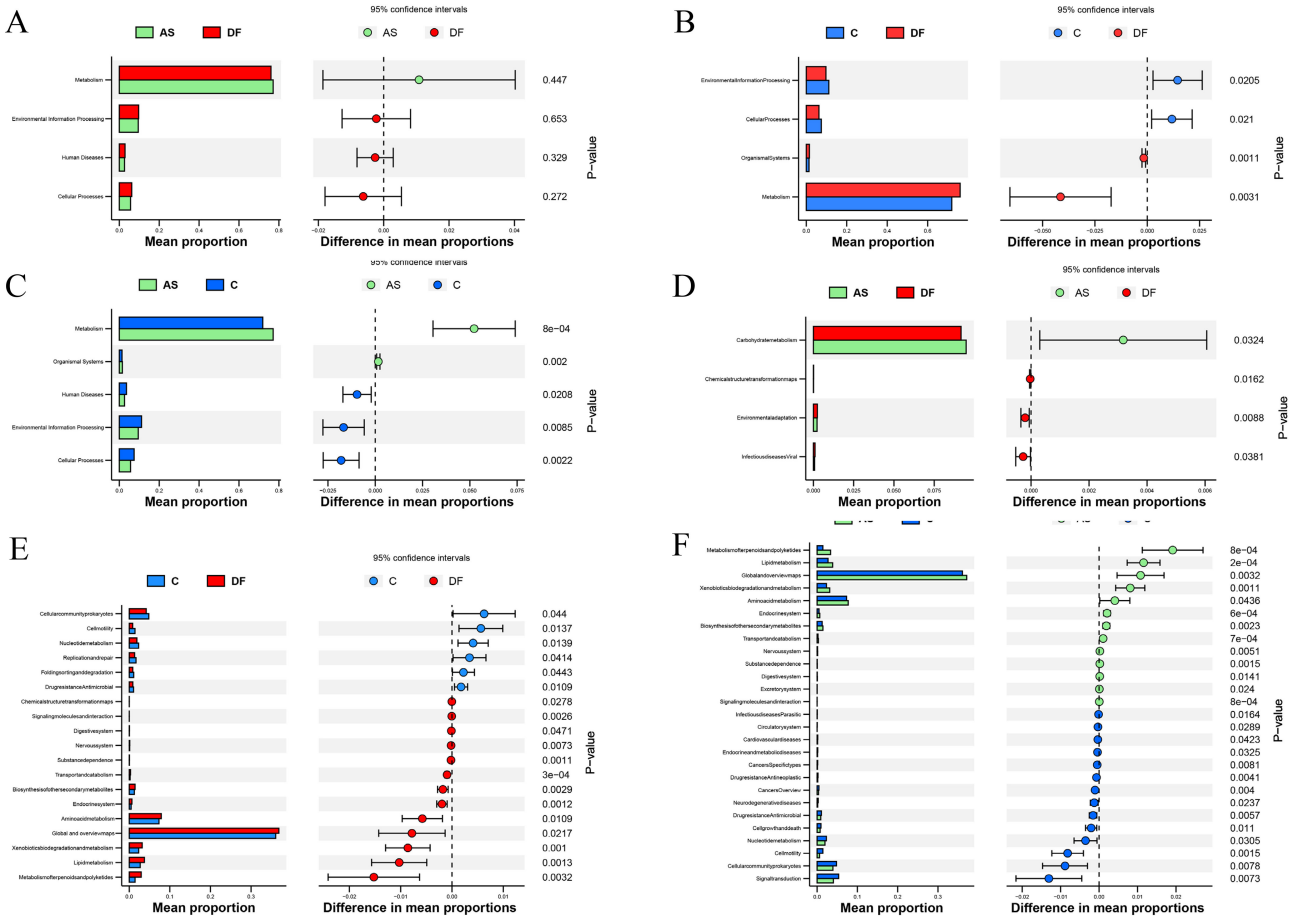
KEGG functional annotation analysis. KEGG functional annotation analysis identified metabolic pathways with significant intergroup differences. The left horizontal bar plot represents the relative abundance of enriched metabolic pathways as a percentage of total metabolic pathways in each group, while the right panel displays the corrected P-values. **(A)** No significant differences between DF and AS at level 1. **(B)** Significant functional differences between DF and C at level 1. **(C)** Significant functional differences between AS and C at level 1. **(D)** Significant functional differences between DF and AS at level 2. **(E)** Significant functional differences between DF and C at level 2. **(F)** Significant functional differences between AS and C at level 2.

At level 1, no significant differences were observed between the DF and AS groups (**Figure 6A**), but both the DF and AS groups showed significant differences compared to the C group (**Figures 6B, C**) (*P* < 0.05). Compared to the C group, both the DF and AS groups exhibited upregulation in Metabolism and Organismal Systems, and downregulation in Cellular Processes and Environmental Information Processing.

At level 2, comparing the DF group with the AS group (**Figure 6D**), significant upregulation was observed in the Carbohydrate metabolism pathway, while Environmental adaptation, Infectious diseases: Viral, and Chemical structure transformation maps pathways were significantly downregulated. Compared to the C group (**Figure 6E**), the DF group showed significant upregulation in pathways such as Global and overview maps, Amino acid metabolism, Lipid metabolism, Xenobiotics biodegradation and metabolism, Metabolism of terpenoids and polyketides, Biosynthesis of other secondary metabolites, and Endocrine system, while significant downregulation was observed in Cellular community – prokaryotes, Nucleotide metabolism, Replication and repair, Drug resistance: Antimicrobial, Folding, sorting and degradation, and Cell motility. Comparing the AS group with the C group (**Figure 6F**), significant upregulation was found in Global and overview maps, Amino acid metabolism, Lipid metabolism, Metabolism of terpenoids and polyketides, Xenobiotics biodegradation and metabolism, Biosynthesis of other secondary metabolites, and Endocrine system, while significant downregulation occurred in Signal transduction, Cellular community – prokaryotes, Nucleotide metabolism, Drug resistance: Antimicrobial, Cell growth and death, Cell motility, Cancers: Overview, and Drug resistance: Antineoplastic.

**Figure 7:**
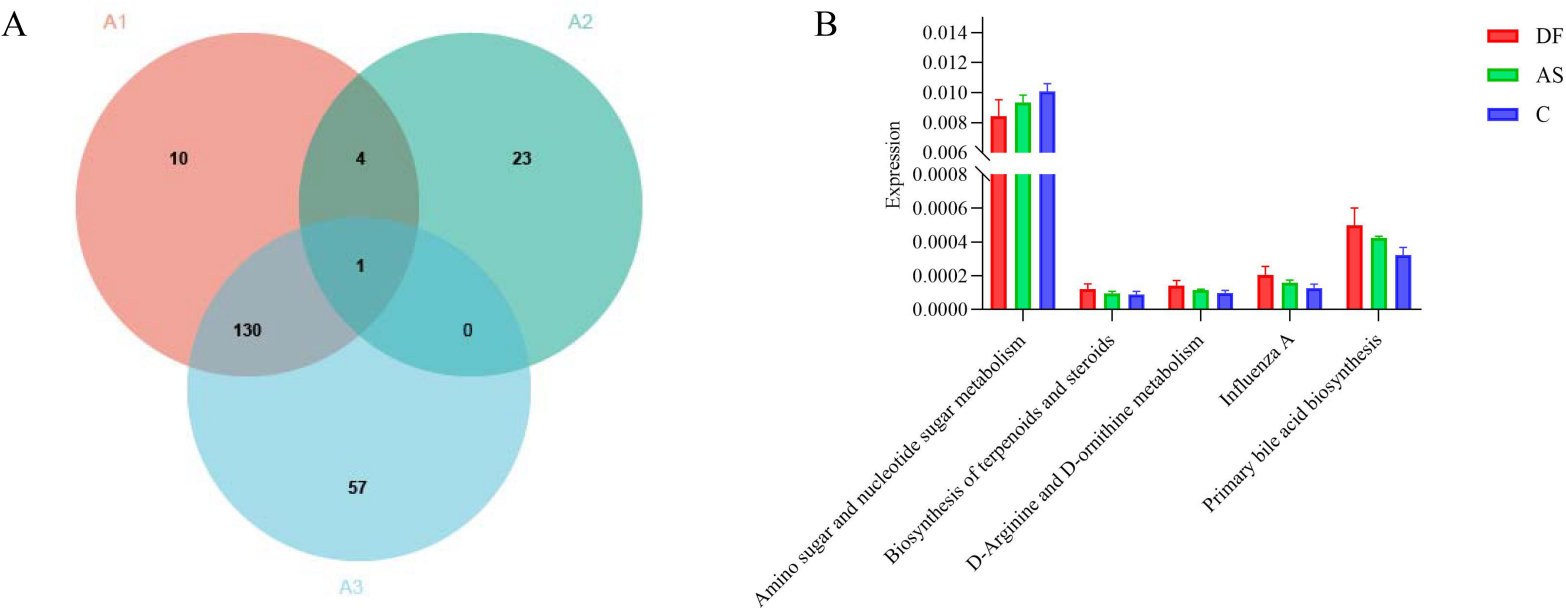
KEGG functional annotation analysis. **(A)** Number of significantly different functions at level 3: (A1) DF vs. C group. (A2) DF vs. AS group. (A3) AS vs. C group. **(B)** Intersection of differentially expressed genes between DF vs. C group and DF vs. AS group.

At level 3 (**Figure 7A**), we identified 28 differential pathways between the DF and AS groups, 145 differential pathways between the DF and C groups, and 188 differential pathways between the AS and C groups. To further explore the role of the microbiota in arterial tissue during the development of diabetic foot ulcers, we conducted a more in-depth analysis at level 3. The results revealed that primary bile acid biosynthesis showed differential expression in both the DF and AS groups compared to the C group. Notably, in contrast to the AS and C groups, the DF group exhibited significant changes in pathways such as amino sugar and nucleotide sugar metabolism, biosynthesis of terpenoids and steroids, D-arginine and D-ornithine metabolism, Influenza A, and primary bile acid biosynthesis (**Figure 7B**). Among these five pathways, amino sugar and nucleotide sugar metabolism and primary bile acid biosynthesis exhibited particularly significant changes.

These findings, together with the increased metabolic pathways observed at levels 1 and 2, suggest that the upregulation of primary bile acid biosynthesis may play a crucial role in the pathogenesis and progression of diabetic foot ulcers. The significant alteration of this pathway in the DF group highlights its potential involvement in modulating metabolic processes that are critical for ulcer development, thus offering valuable insights into possible therapeutic targets for managing diabetic foot ulcers.

## Discussion

Dysbiosis is closely related to the development of diseases. This study provides preliminary, exploratory insights into the composition and potential roles of arterial microbiota in diabetic foot and, for the first time, compares the arterial tissue microbiota of DF patients with that of AS patients and healthy controls who underwent amputation due to trauma, to explore the potential link between the microbiota and diabetic foot. The results showed that the microbial communities in the arterial tissue of DF patients shared certain similarities with those of AS patients. At the phylum level, the abundance of Actinobacteria was significantly increased in both the DF and AS groups, while the abundance of TM6 was significantly reduced in the DF group and the abundance of Proteobacteria was significantly reduced in the AS group. Further LEfSe analysis revealed that the primary representative genera in the DF group were *Corynebacterium*, *Streptophyta_Group*, *Caulobacter*, *Hydrogenophaga*, and *Diaphorobacter*. KEGG pathway analysis further revealed functional pathway differences among the groups. At level 1, no significant differences were observed between the DF and AS groups, but both groups exhibited upregulation in metabolism compared to the C group. At level 2, compared to the C group, both the DF and AS groups showed significant upregulation in pathways such as global and overview maps, amino acid metabolism, and lipid metabolism. Notably, primary bile acid biosynthesis showed differences between the DF, AS, and C groups. Additionally, distinct from the AS and C groups, the DF group exhibited significant changes in pathways such as amino sugar and nucleotide sugar metabolism, biosynthesis of terpenoids and steroids, D-arginine and D-ornithine metabolism, and primary bile acid biosynthesis.

Our results show that the Alpha diversity of DF patients is lower than that of AS patients and significantly lower than that of healthy controls, suggesting that dysbiosis plays an important role in the onset and progression of diabetic foot (Gardiner et al., 2017). Further analysis of the microbial community composition revealed that the predominant phyla in the arteries of DF patients include Firmicutes, Proteobacteria, Actinobacteria, and Bacteroidota, which are similar to the major microbiota in the skin of diabetic foot ulcer sites (Pang et al., 2020). However, there is still controversy regarding the microbial composition in diabetic foot patients. Studies by Mengchen Zou and Yaoming Xue (Zhang et al., 2023) found that the abundance of increased, while the abundance of Proteobacteria and Bacteroidota decreased in DF patients. On the other hand, Biao Cheng (Pang et al., 2020) and colleagues observed an increase in the abundance of Actinobacteria in DF patients. These findings partially align with our results, where Firmicutes, Proteobacteria, and Bacteroidota were the predominant phyla in DF patients, but their abundance did not differ significantly from that in the control group. Notably, the abundance of *Actinobacteria* increased in the DF group. Given that local sampling in diabetic foot ulcers might be influenced by sampling methods and contamination, which contribute to the variability in previous studies, these findings highlight the importance of exploring microbiota changes in the arteries of diabetic foot patients. Since the pathological processes of diabetic foot share similarities with atherosclerosis, we included AS patients as a control group for a more precise analysis. The results showed that AS patients also exhibited a significant increase in the abundance of Actinobacteria. As is well known, diabetic foot and atherosclerosis share significant pathological similarities (Deng et al., 2023; Ding et al., 2022). Based on these results, we hypothesize that may not be specific to the pathogenesis of diabetic foot but could be related to the occurrence of arterial atherosclerotic lesions. That is, changes in the abundance of Actinobacteria may reflect alterations in the vascular pathological state (Gao et al., 2022; Lindskog Jonsson et al., 2017; Shi et al., 2024), rather than being solely associated with diabetic foot. This finding further supports our view that changes in the vascular microbiota are likely closely related to the pathological process of atherosclerosis.

LEfSe analysis further identified biomarkers related to diabetic foot. By comparing the microbial composition in the arteries of DF, AS, and C groups, we found that genera such as *Corynebacterium*, *Streptophyta_Group*, *Caulobacter*, *Hydrogenophaga*, and *Diaphorobacter* were significantly enriched in DF patients. These genera may be associated with the development and progression of diabetic foot ulcers. Previous studies have shown that *Corynebacterium* can inhibit the healing of diabetic foot ulcers (DFUs) by promoting inflammation, apoptosis, and pyroptosis (Zheng et al., 2024), and its strong biofilm formation properties may contribute to chronic ulcers (Mottola et al., 2016). However, the roles of other genera such as *Streptophyta_Group*, *Caulobacter*, *Hydrogenophaga*, and *Diaphorobacter* in diabetic foot remain unexplored, and their specific impacts on inflammation and the course of DFUs still need to be investigated. Recent studies suggest that dysbiosis, by increasing the proportion of harmful microbiota and their metabolic products, activates the host immune system, triggering persistent inflammatory responses (Voronina and Arapidi, 2024). This immune imbalance not only weakens the host’s antimicrobial functions but also exacerbates local ischemia and infection through biofilm formation (Lanter et al., 2014; Pouget et al., 2020), further worsening the course of diabetic foot (Macdonald et al., 2021; Muhlestein, 2000; Ross, 1999). Other studies have shown that pathogenic bacteria such as significantly increase at ulcer sites in diabetic patients (Redel et al., 2013), and their relative abundance is associated with ulcer severity and duration (Pouget et al., 2020). Additionally, specific bacteria like can infect macrophages and promote lipid droplet accumulation, accelerating the formation of atherosclerosis (Khan et al., 2014). Notably, microbiota may also have an anti-inflammatory effect in some cases (Yoshida et al., 2018), suggesting that the role of microbiota in disease is complex and dual. In conclusion, research on the microbial composition and function in diabetic foot remains limited. In this study, trauma patients undergoing amputation were used as healthy controls. Acute trauma could potentially induce stress-related changes in microbial communities, but evidence on its effects in lower limb arteries is limited. Studies on gut microbiota suggest that compositional shifts may occur around 72 hours post-trauma, highlighting a direction for future research on vascular microbial communities. In addition,our study also included AS patients as a non-DF comparison group, which helped to further distinguish microbial features potentially unique to diabetic foot ulcers. Further exploration of the inflammatory roles of specific microbiota and their potential impact on DFUs progression will help uncover the pathogenesis of diabetic foot and provide scientific evidence for targeted therapies. Diabetic foot ulcers are difficult to heal and have a high risk of recurrence (Schneider et al., 2021). The exact mechanisms remain unclear. Although significant progress has been made in recent decades with novel antibiotics, surgical resection, negative pressure wound therapy (NPWT), and other treatment methods, the prognosis remains poor due to the rapid spread of infections and overwhelming tissue damage, which often leads to amputation (Lipsky et al., 2020). To explore the potential role and mechanisms of microbiota in the progression of diabetic foot, we performed a hierarchical analysis of KEGG functional pathways. PICRUSt2-based predictions, such as primary bile acid biosynthesis, provide valuable preliminary insights into potential microbial functions in arterial tissues. While these predictions are based on 16S rRNA gene sequencing and should be interpreted cautiously, the NSTI scores suggest reasonable accuracy. Experimental validation through targeted metabolomics would further substantiate these functional predictions. The level 1 and 2 functional pathways indicate that the microbial communities in the arteries of diabetic foot patients and AS patients significantly upregulate amino acid metabolism and lipid metabolism, suggesting that these metabolic pathways may play a role in regulating inflammation and disease progression. The role of amino acid metabolism in immune and inflammatory responses is widely recognized (Sun et al., 2022). Studies have shown that abnormal amino acid metabolism is closely associated with insulin resistance (Guasch-Ferré et al., 2016) and the progression of atherosclerosis (Zhao et al., 2023). Disruption in branched-chain amino acid metabolism can lead to endothelial dysfunction by increasing reactive oxygen species and inflammation (Zhenyukh et al., 2018), while abnormal phenylalanine metabolism can induce pro-inflammatory macrophage polarization (Lv et al., 2023). Similarly, abnormal lipid metabolism leads to oxidative lipid accumulation and endoplasmic reticulum stress (Zhu et al., 2019), further activating inflammation. Chronic inflammation is not only a key factor in atherosclerosis development but also closely related to diabetes (Poznyak et al., 2020). In addition, dysbiosis as a trigger for chronic inflammation has attracted widespread attention (Eshghjoo et al., 2021). The enrichment of amino acid and lipid metabolism pathways in the arterial microbiota of diabetic foot and atherosclerosis patients suggests that microbiota may regulate these metabolic pathways, inducing inflammation and contributing to disease progression.

To further understand the disease progression of diabetic foot, we conducted a more detailed analysis of the KEGG pathways at level 3. The results showed significant downregulation in the Amino sugar and nucleotide sugar metabolism pathway and significant upregulation in Primary bile acid biosynthesis. As a key branch of carbohydrate metabolism, Amino sugar and nucleotide sugar metabolism is responsible for the synthesis, conversion, and degradation of amino sugars and nucleotide sugars. Its downregulation is closely related to carbohydrate metabolism disorders in diabetic patients and is not the primary mechanism for the delayed healing of diabetic foot ulcers due to dysbiosis. Furthermore, level 1 and 2 functional pathway analyses indicate the upregulation of overall metabolism and lipid metabolism in diabetic foot patients, supporting the downregulation of Amino sugar and nucleotide sugar metabolism as the main functional pathway influencing ulcer healing in non-diabetic foot patients. Primary bile acid biosynthesis is an important branch of lipid metabolism and another significantly enriched metabolic pathway in the level 3 analysis. The key products of bile acid metabolism, cholic acid and chenodeoxycholic acid, have been found to be closely associated with inflammation (Chiang, 2013; Dabke et al., 2019; Guan et al., 2022). Notably, chenodeoxycholic acid can activate the NLRP3 inflammasome (Hao et al., 2017), inducing systemic chronic low-grade inflammation. The upregulation of may exacerbate chronic inflammation and inhibit local tissue repair, thereby hindering the healing of diabetic foot ulcers. We recognize that the modest cohort size, particularly the limited availability of control samples, may constrain the robustness of our conclusions. This reflects the inherent challenges in obtaining well-matched clinical specimens; nevertheless, the statistically significant results obtained here still provide important mechanistic insights. Meanwhile, the clinical relevance of arterial microbiota findings remains unclear due to limited evidence, and further studies are needed.

## Conclusion

Combining the hierarchical analysis of the above functional pathways, we speculate that the abnormal upregulation of Primary bile acid biosynthesis may be linked to delayed healing of diabetic foot ulcers, potentially exacerbating chronic inflammation and local tissue damage. This observation highlights a promising avenue for future investigation. Targeted modulation of the rate-limiting steps in Primary bile acid biosynthesis could represent a novel therapeutic strategy for diabetic foot ulcers by mitigating inflammation and improving local vascular function, thereby promoting ulcer healing. These findings provide a potential intervention target for the treatment of diabetic foot.

## Data Availability

The original contributions presented in the study are included in the article/**Supplementary Material**. Further inquiries can be directed to the corresponding author.
