# Supplementary material for: Increased abundance of actinobacteria and upregulation of primary bile acid biosynthesis in diabetic foot ulcers
Source: Front Cell Infect Microbiol. 2025 Nov 20;15:1651828. doi: 10.3389/fcimb.2025.1651828 (PMC12675428; doi:10.3389/fcimb.2025.1651828)
Supplement: Supplementary file 1 [file Table1.doc]

**Supplementary Table 1. Summary of Sequencing Data Quality for All Sample**

| **Sample** | **Raw Data** | **Clean Data** | **Clean%** | **Q20%** | **Q30%** | **GC%** |
| --- | --- | --- | --- | --- | --- | --- |
| **DF1** | 204139 | 184820 | 90.54 | 97.64 | 96.30 | 56.70 |
| **DF2** | 211110 | 188684 | 89.38 | 97.74 | 96.35 | 57.07 |
| **DF3** | 210010 | 196525 | 93.58 | 97.82 | 96.50 | 57.50 |
| **DF4** | 202612 | 177663 | 87.69 | 98.06 | 94.22 | 56.35 |
| **DF5** | 204292 | 180316 | 88.26 | 98.28 | 94.67 | 56.65 |
| **DF6** | 214892 | 194138 | 90.34 | 98.16 | 94.50 | 56.35 |
| **DF7** | 197796 | 184046 | 93.05 | 98.27 | 94.88 | 55.22 |
| **DF8** | 165823 | 146062 | 88.08 | 98.37 | 95.19 | 55.98 |
| **DF9** | 214004 | 192168 | 89.80 | 96.28 | 94.44 | 56.65 |
| **DF10** | 219810 | 194806 | 88.62 | 96.98 | 95.30 | 57.03 |
| **DF11** | 208026 | 193714 | 93.12 | 97.05 | 95.40 | 57.47 |
| **AS1** | 212776 | 200049 | 94.02 | 97.85 | 96.44 | 55.97 |
| **AS2** | 209949 | 190080 | 90.54 | 97.71 | 96.34 | 56.08 |
| **AS3** | 200019 | 179440 | 89.71 | 97.67 | 96.27 | 56.39 |
| **AS4** | 195492 | 181411 | 92.80 | 98.03 | 94.43 | 56.17 |
| **AS5** | 202317 | 189279 | 93.56 | 97.25 | 95.58 | 55.94 |
| **AS6** | 218667 | 196453 | 89.84 | 96.55 | 94.77 | 56.03 |
| **AS7** | 211251 | 188158 | 89.07 | 96.55 | 94.76 | 56.35 |
| **C1** | 190371 | 165046 | 86.70 | 97.73 | 93.83 | 57.06 |
| **C2** | 181787 | 155872 | 85.74 | 98.05 | 94.65 | 54.39 |
| **C3** | 193215 | 169281 | 87.61 | 98.07 | 94.49 | 55.81 |
| **C4** | 192317 | 178170 | 92.64 | 98.45 | 95.35 | 54.70 |

**Supplementary Table 2. Representative Phylum-Level Differences Across Groups**

|  | *p(DF/C)* | *p(AS/C)* | *p(DF/AS)* |
| --- | --- | --- | --- |
| Proteobacteria | 0.091 | 0.020 | 0.514 |
| Firmicutes | 0.734 | 0.307 | 0.358 |
| Actinobacteria | 0.009 | 0.003 | 0.608 |
| Bacteroidetes | 0.837 | 0.442 | 0.223 |
| OD1 | 0.349 | 0.477 | 0.745 |
| TM6 | 0.011 | 0.194 | 0.318 |

**Supplementary Table 3. ANOSIM R statistic**

| group | R | *p* |
| --- | --- | --- |
| DF/AS | -0.023 | 0.525 |
| DF/C | 0.295 | 0.009 |
| AS/C | 0.698 | 0.010 |

**Supplementary Table 4. LDA scores of significant taxa identified by LEfSe analysis**

| Name | Group | LDA | *p* |
| --- | --- | --- | --- |
| Corynebacterium | DF | 3.657 | 0.005 |
| Streptophyta_Group | DF | 3.286 | 0.015 |
| Caulobacter | DF | 3.174 | 0.029 |
| Hydrogenophaga | DF | 3.151 | 0.013 |
| Diaphorobacter | DF | 3.057 | 0.041 |
| Bacteroides | AS | 4.431 | 0.006 |
| Xenorhabdus | AS | 4.183 | 0.027 |
| Escherichia_Shigella | AS | 3.870 | 0.043 |
| Anaerococcus | AS | 3.818 | 0.034 |
| Finegoldia | AS | 3.519 | 0.049 |
| Cronobacter | AS | 3.471 | 0.028 |
| Ruminococcaceae_Group | AS | 3.445 | 0.034 |
| Unclassified_Streptophyta | AS | 3.344 | 0.016 |
| Roseburia | AS | 3.143 | 0.026 |
| Ruminococcus | AS | 3.066 | 0.014 |
| Prevotella | C | 4.279 | 0.020 |
| Serratia | C | 3.915 | 0.041 |
| Mycoplana | C | 3.626 | 0.037 |
| vadinBC27 | C | 3.611 | 0.047 |
| Chelonobacter | C | 3.521 | 0.039 |
| Lysinibacillus | C | 3.472 | 0.009 |
| Marinomonas | C | 3.470 | 0.035 |
| Unclassified_SJA_4 | C | 3.380 | 0.026 |
| Bulleidia | C | 3.348 | 0.005 |
| Selenomonas | C | 3.101 | 0.037 |
| Granulicatella | C | 3.047 | 0.042 |
